# Systematic bibliometric and visualized analysis of research hotspots and trends in artificial intelligence in autism spectrum disorder

**DOI:** 10.3389/fninf.2023.1310400

**Published:** 2023-12-06

**Authors:** Qianfang Jia, Xiaofang Wang, Rongyi Zhou, Bingxiang Ma, Fangqin Fei, Hui Han

**Affiliations:** ^1^Hebei University of Chinese Medicine, Shijiazhuang, China; ^2^Children’s Brain Disease Diagnosis, Treatment and Rehabilitation Center of the First Affiliated Hospital of Henan University of Chinese Medicine, Zhengzhou, China; ^3^School of Pediatric Medicine, Henan University of Chinese Medicine, Zhengzhou, China; ^4^Department of Nursing, the First People’s Hospital of Huzhou, Huzhou University, Huzhou, China

**Keywords:** artificial intelligence, autism spectrum disorder, data visualization, bibliometric, CiteSpace, VOSviewer

## Abstract

**Background:**

Artificial intelligence (AI) has been the subject of studies in autism spectrum disorder (ASD) and may affect its identification, diagnosis, intervention, and other medical practices in the future. Although previous studies have used bibliometric techniques to analyze and investigate AI, there has been little research on the adoption of AI in ASD. This study aimed to explore the broad applications and research frontiers of AI used in ASD.

**Methods:**

Citation data were retrieved from the Web of Science Core Collection (WoSCC) database to assess the extent to which AI is used in ASD. CiteSpace.5.8. R3 and VOSviewer, two online tools for literature metrology analysis, were used to analyze the data.

**Results:**

A total of 776 publications from 291 countries and regions were analyzed; of these, 256 publications were from the United States and 173 publications were from China, and England had the largest centrality of 0.33; Stanford University had the highest H-index of 17; and the largest cluster label of co-cited references was machine learning. In addition, keywords with a high number of occurrences in this field were autism spectrum disorder (295), children (255), classification (156) and diagnosis (77). The burst keywords from 2021 to 2023 were infants and feature selection, and from 2022 to 2023, the burst keyword was corpus callosum.

**Conclusion:**

This research provides a systematic analysis of the literature concerning AI used in ASD, presenting an overall demonstration in this field. In this area, the United States and China have the largest number of publications, England has the greatest influence, and Stanford University is the most influential. In addition, the research on AI used in ASD mostly focuses on classification and diagnosis, and “infants, feature selection, and corpus callosum are at the forefront, providing directions for future research. However, the use of AI technologies to identify ASD will require further research.

## 1 Introduction

Artificial intelligence (AI) was originally described in 1950 as the concept of using computers to model intelligent behavior and critical thinking (Kaul et al., 2020). With the development of AI technologies, AI systems are now able to analyze complex algorithms and self-learning. Therefore, AI is beginning to be widely used in medical research and clinical practice (Kulkarni et al., 2020). In medicine, use of AI has exponentially enhanced predictive analyses and image recognition (Reddy, 2022). Many clinical assistant systems have been proposed by researchers since the mid-twentieth century (Hamet and Tremblay, 2017). AI has been studied for screening, diagnosis, and intervention in autism spectrum disorder (ASD), and several system models have been proposed (Shahamiri and Thabtah, 2020; Ahmed et al., 2022; Barua et al., 2022; Kanhirakadavath and Chandran, 2022). The position of AI in the identification and diagnosis of ASD based on neuroimaging data is becoming increasingly important (Bilgen et al., 2020; Pan et al., 2021; Duan et al., 2022). And according to the published reports, AI may have a profound effect on ASD in the coming decades, (Mertz, 2021; Alabdulkareem et al., 2022).

Bibliometric measurements have been utilized in earlier studies to analyze ASD (Ernesto Martinez-Gonzalez and Andreo-Martinez, 2022; Li et al., 2022; Lu et al., 2022; Jiang et al., 2023). The topics of these studies were about ASD alone or ASD merged with other fields, however, research on the utilization of AI in ASD is limited. Specially, most of them were reviews of a specific technology for ASD applications; comprehensive studies of AI technology in ASD applications are lacking. This study intended to provide a thorough overview of the broad applications and research frontiers of the use of AI in ASD by examining multiple aspects. Publications on AI in ASD from Web of Science Core Collection (WoSCC) database were examined through bibliometric approaches. A key aspect of this study is to elaborate a reproducible and impartial approach to exploring the active research frontiers. Specifically, we explored the current applications of applied AI, upcoming advancements, and possible obstacles to ASD. This paper is expected to be a useful source for AI specialists, diagnosticians, physicians and medical imaging researchers.

## 2 Materials and methods

**Figure 1:**
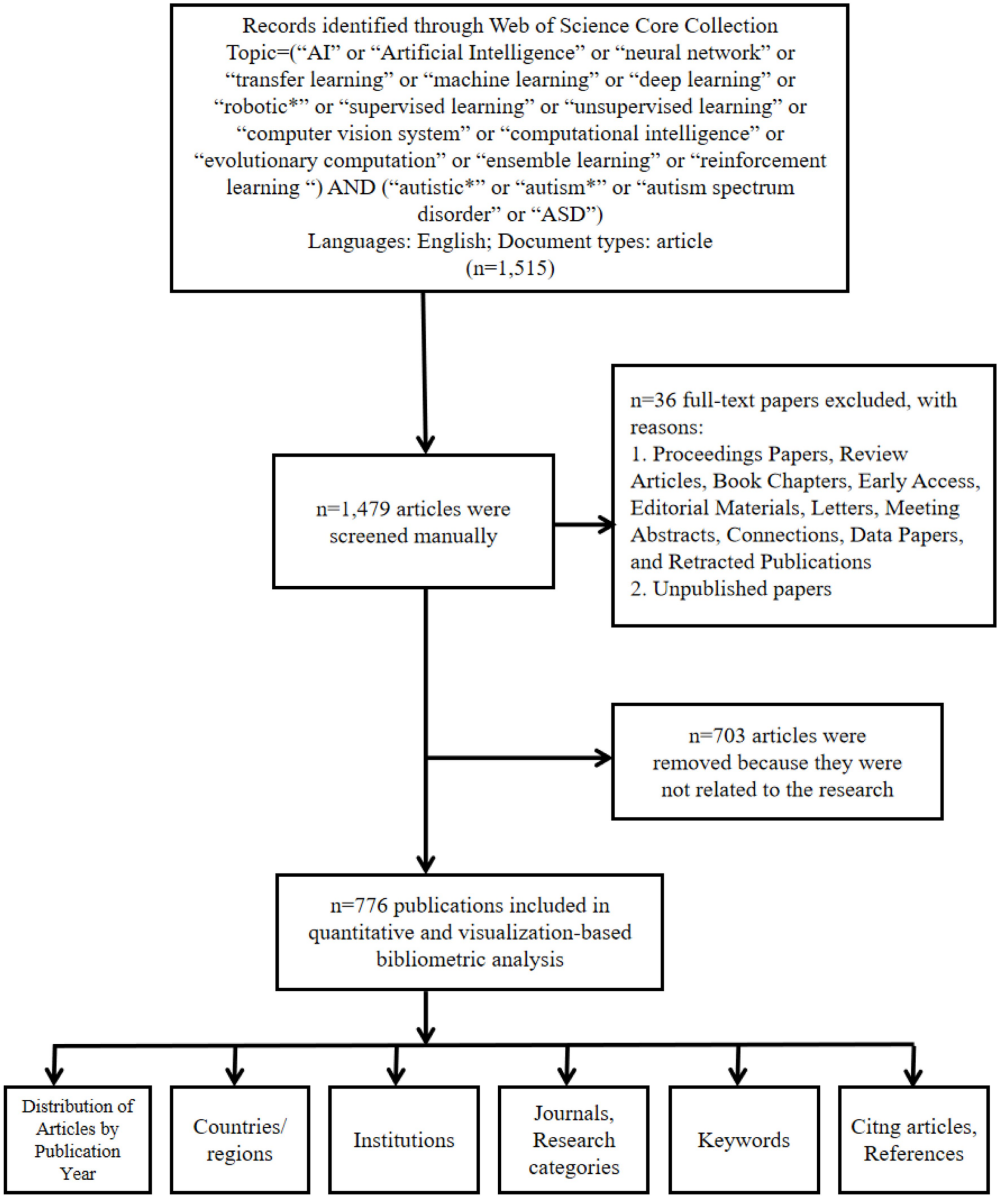
Framework flow diagram showing the detailed selection criteria and bibliometric analysis steps for the study of AI in ASD. AI, artificial intelligence; ASD, autism spectrum disorder.

On September 1, 2023, published citation data were retrieved from Web of Science Core Collection (WoSCC) database. Two authors certified the data separately (Qianfang Jia and Xiaofang Wang). The formula used to search was TS = (“autistic∗” or “autism∗” or “ASD” or “autism spectrum disorder”) AND (“AI” or “Artificial Intelligence” or “neural network” or “transfer learning” or “machine learning” or “deep learning” or “robotic*” or “supervised learning” or “unsupervised learning” or “computer vision system or computational intelligence or evolutionary computation or ensemble learning or reinforcement learning). The search was limited to English-language literature and articles. After reading the titles and abstracts of each publication, we manually excluded data to obtain the most reliable results. Requirements for manual exclusion included the following: (1) The study’s field excluded medicine, (2) The study did not employ AI techniques, and (3) the study was regarding another disease than ASD. All data included in the analysis refer to research on AI in ASD. Figure 1 depicts the exhaustive search and analysis processes.

CiteSpace.5.8. R3 and VOSviewer were used to evaluate collaborative networks of countries, regions, institutions, journals, keywords, references, and research categories. The number of publications supplied by countries or regions and by year were counted using the WoSCC database’s citation analyzer, and the cooperative relationships between countries and regions were evaluated together with the number of citations using CiteSpace’s default settings. This article describes all citation characteristics.

## 3 Results

### 3.1 Distribution of articles by publication year

**Figure 2:**
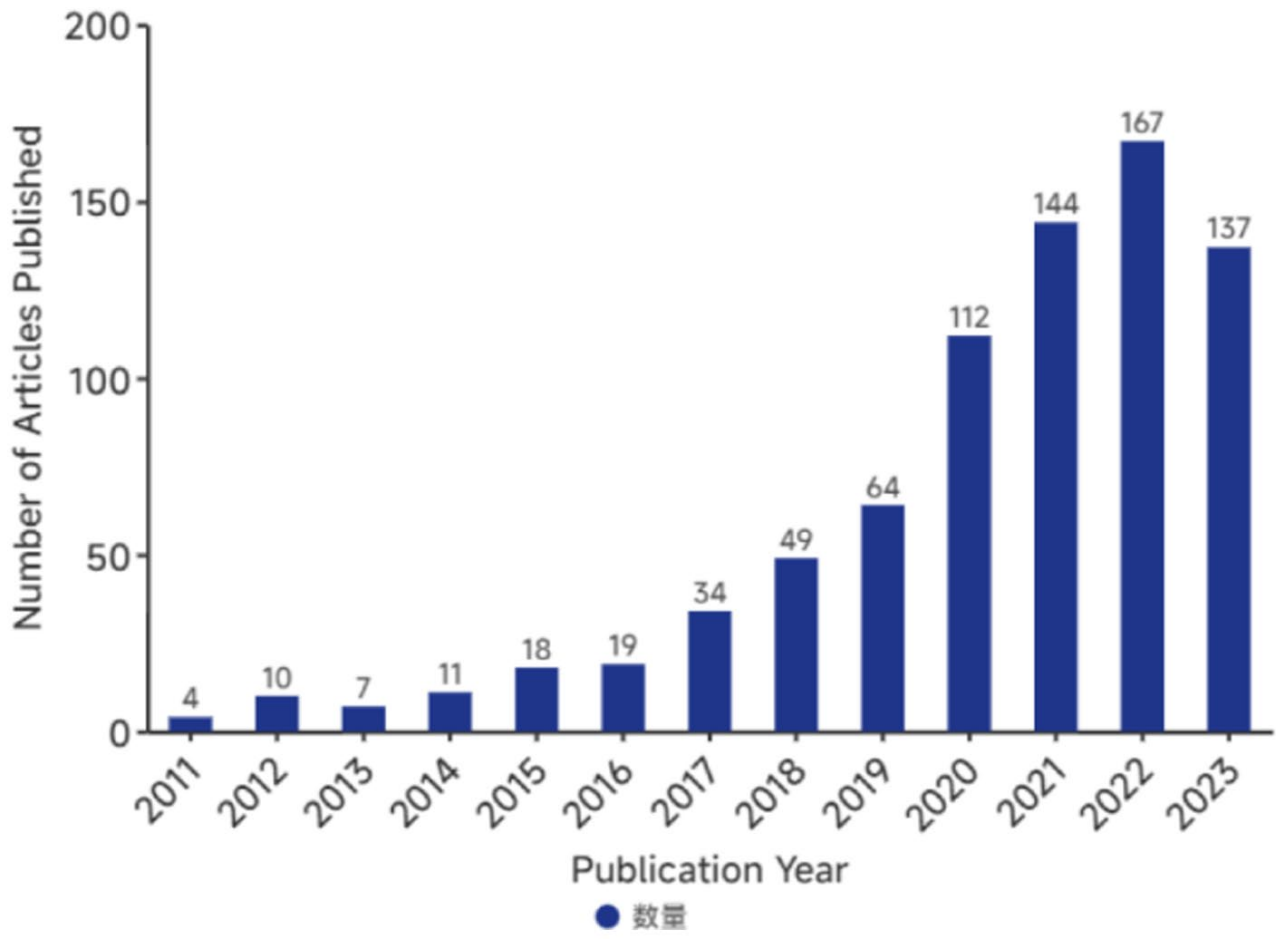
Annual number of publications on AI in ASD. AI, artificial intelligence; ASD, autism spectrum disorder.

This research retrieved 796 publications that focused on use of AI in ASD. The number of articles published annually during this period is shown in Figure 2. We did not restrict the time of publication of the literature; however, the included articles had a publication period of 2011–2023. Since 2020, the annual number of articles regarding AI in ASD has exceeded one hundred and has increased rapidly in recent years (The number of articles in 2023 is lower than in 2022 as the search date for 2023 ends in September).

### 3.2 Countries or regions

**Figure 3:**
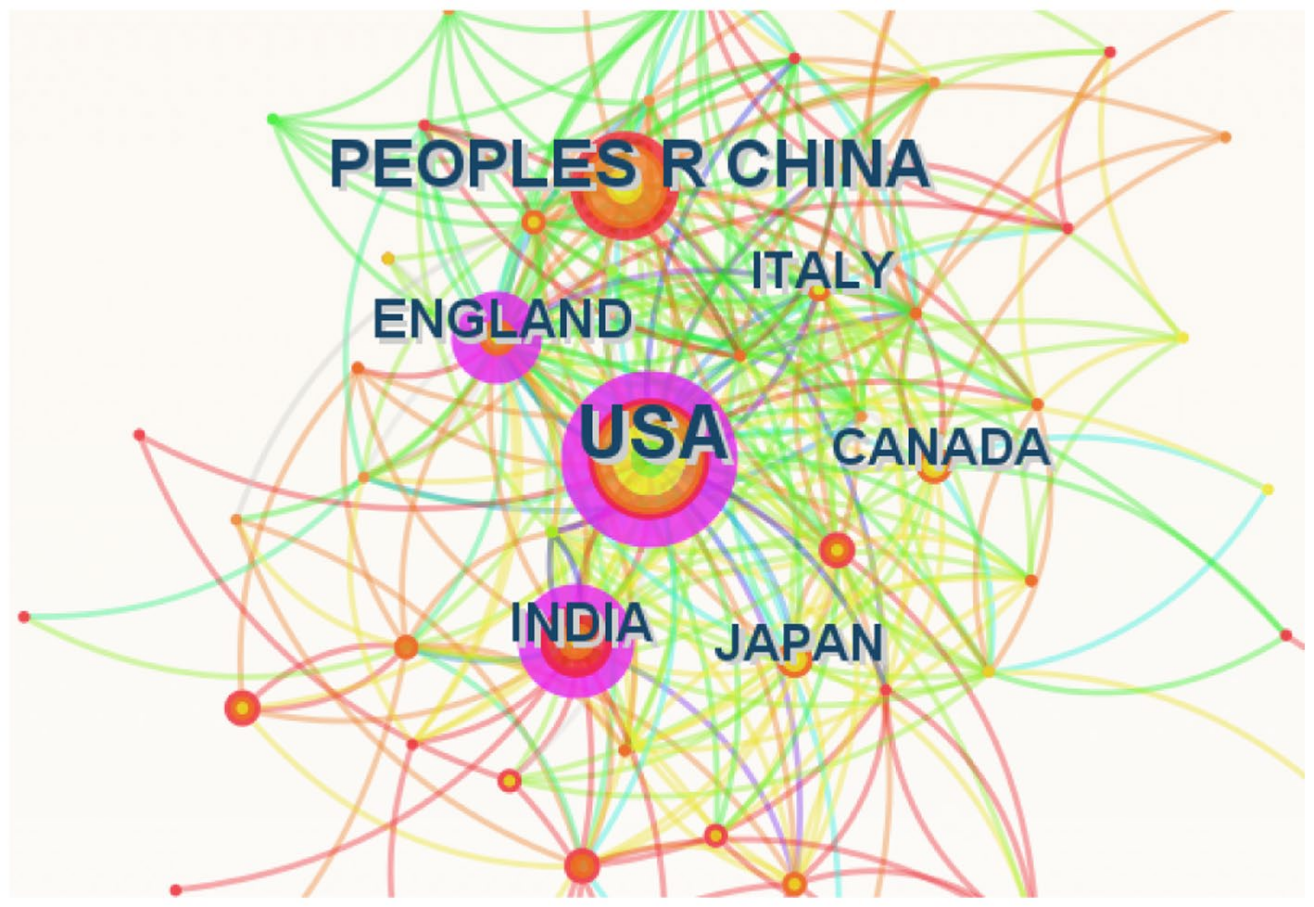
Keywords with the strongest citation bursts for publications on AI in ASD. AI, artificial intelligence; ASD, autism spectrum disorder; fMRI, functional magnetic resonance imaging.

The publications covered 291 countries and regions. The size of each label and node area in Figure 3 indicate the total number of publications by country or region. The United States (256 articles), the People’s Republic of China (173 articles), and India (82 articles) are among the countries having sizable labels and node areas. The cooperation between countries or regions is shown by the connection between the nodes. The influence is stronger in countries or regions with more connections. The purple circle’s area represents the national influence of publications, which is shown in Table 1 as centrality. The area of purple circle in England is the largest (0.33), indicating that publications published in England in the field of AI used in ASD have the most overall influence. These conclusions are substantiated factually in Table 1. Academic achievement can be accurately assessed by the H-index. The influence of a publication increases as a function of its centrality and its H-index. Generally, the United States and China published the largest number of publications, whereas publications from England had the greatest influence.

**Table 1:** Top 10 countries or regions with publications on AI in ASD.

Rank	Countries or regions	Counts	Centrality	H-index
1	United States	256	0.25	48
2	China	173	0.10	22
3	India	82	0.18	13
4	England	72	0.33	26
5	Italy	50	0.01	15
6	Japan	45	0.03	18
7	Canada	43	0.06	16
8	Australia	30	0.06	11
9	Germany	25	0.03	11
10	United Arab Emirates	25	0.04	8

AI, artificial intelligence; ASD, autism spectrum disorder.

### 3.3 Institutions

**Figure 4:**
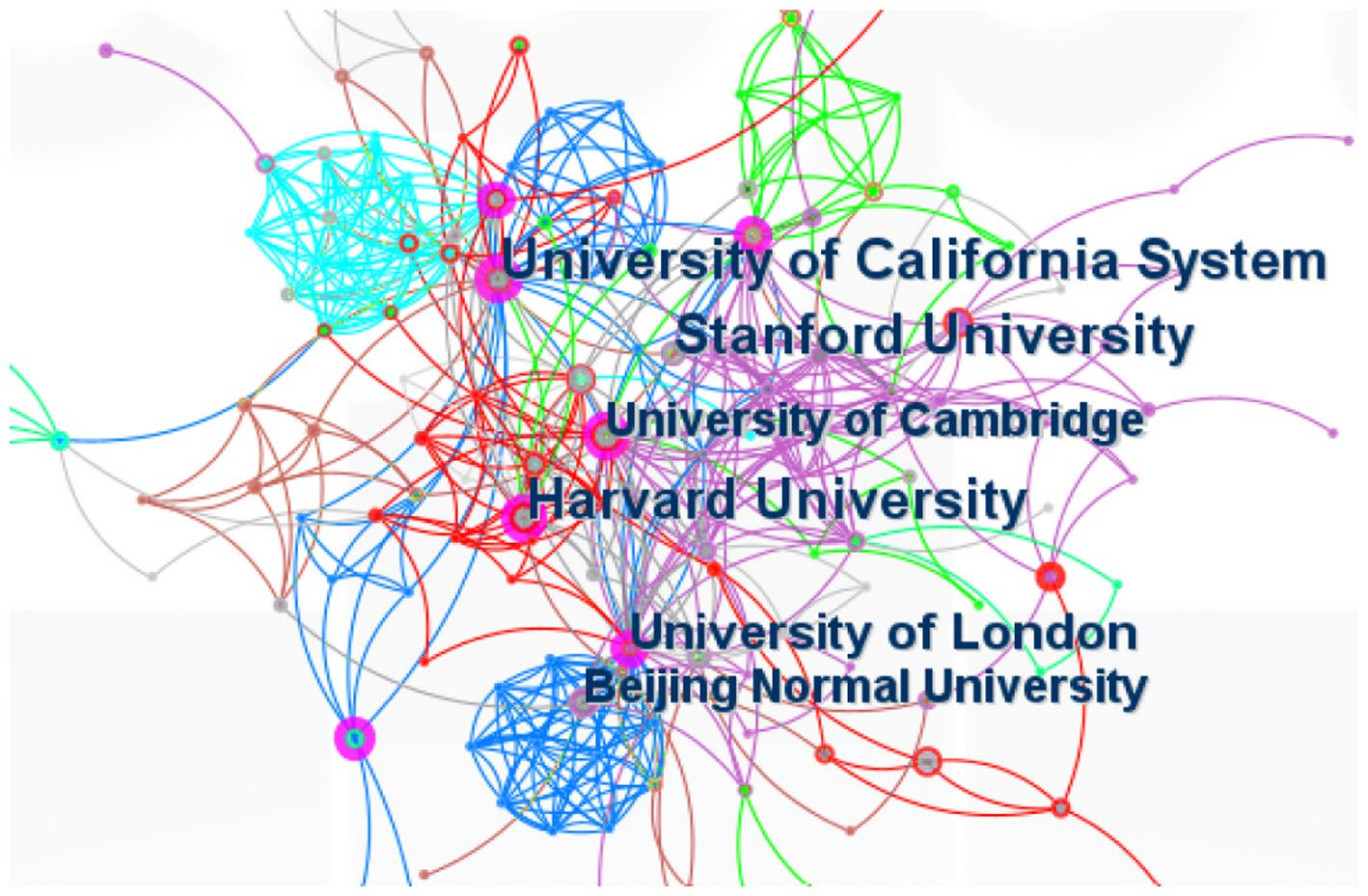
Co-cited reference timeline map of publications on AI in ASD. AI, artificial intelligence; ASD, autism spectrum disorder.

Table 2 displays the top 10 institutions in the number of publications. The data displayed is the result of CiteSpace’s default settings. Stanford University’s H-index of 17 was the highest. Five American institutions, two Chinese institutions, two England institutions, and one Egyptian institution made up the top 10 institutions. All three of the top institutions with the highest H-index were American. All three top-ranked institutions were American. The inter-agency coordination is shown in Figure 4‘s connection between the tags. The number of publications sent by institutions is indicated by the node size.

**Table 2:** Top 10 institutions with publications on AI in ASD.

Rank	Institution	Country or regions	Counts	H-index
1	Harvard University	United States	27	15
2	University of California System	United States	27	15
3	Stanford University	United States	26	17
4	University of London	England	23	10
5	Harvard Medical School	United States	17	11
6	University of Cambridge	England	16	9
7	Beijing Normal University	China	15	6
8	Egyptian Knowledge Bank Ekb	Egypt	14	4
9	Shenzhen University	China	14	5
10	Vanderbilt University	United States	14	10

AI, artificial intelligence; ASD, autism spectrum disorder.

### 3.4 Journals and research categories

**Figure 5:**
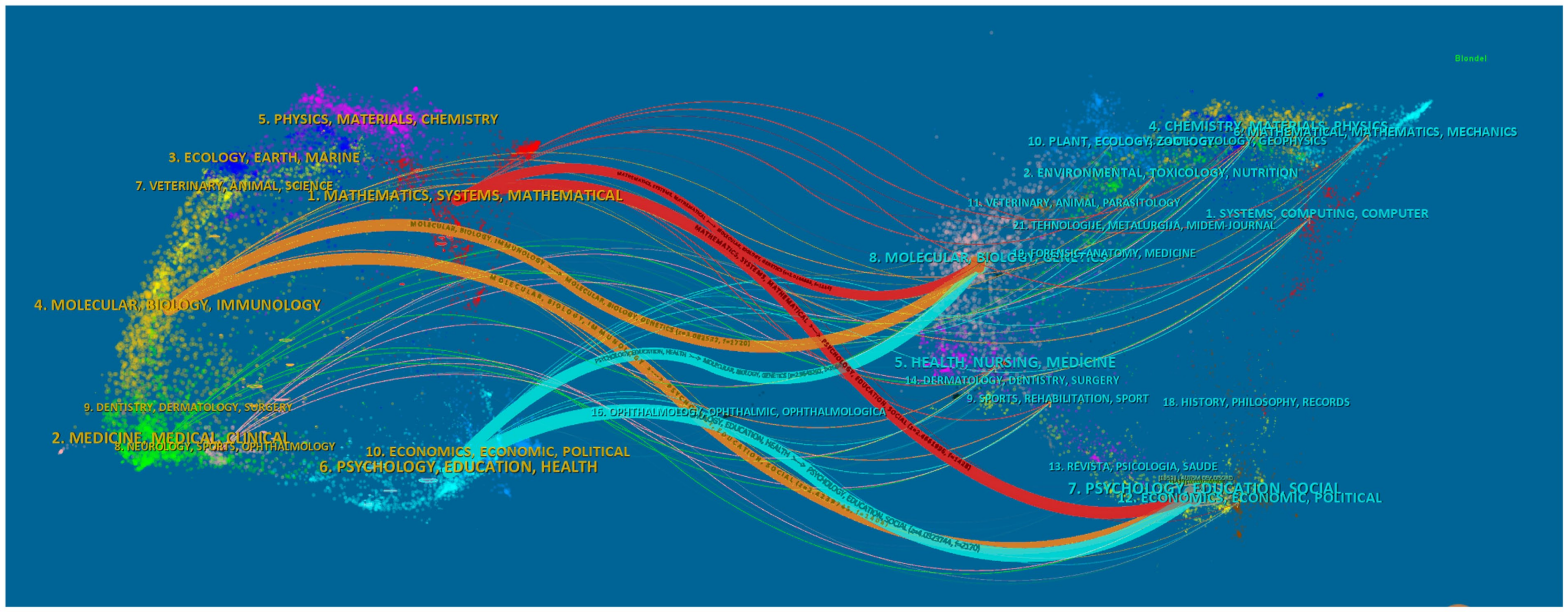
Cooperation between countries or regions that contributed to publications on AI in ASD. AI, artificial intelligence; ASD, autism spectrum disorder. AI, artificial intelligence; ASD, autism spectrum disorder.

Publications in the cited journals provide the knowledge foundation for research in this field. Research presented in highly citing journals indicates current research hotspots. CiteSpace was used to visualize citation relationships. The traces of colored lines in Figure 5 indicate the citation relationships of active research areas. The left and right parts of Figure 5 indicate the research fields of the citing journals and cited journals. The red trace displays a classification of the journals with the largest number of publications. The areas of knowledge-foundation research regarding AI application in ASD include psychology, education, social science, molecular biology, genetics, health, nursing and medicine, comprising the hotspot areas, such as mathematics and mathematical systems. The research categories of the citing and cited journals in the top 10 are presented in Tables 3, 4. The most popular area of research in the citing journals was science and technology. Psychology was the discipline most commonly referred in the cited journals.

**Table 3:** Top 10 citing journals with publications on AI in ASD.

Rank	Citing journals	Research fields	Counts	Journal impact factor 2022
1	Scientific Reports	Science & Technology	32	4.6
2	IEEE Access	Computer Science; Engineering; Telecommunications	29	3.9
3	Frontiers in Psychiatry	Psychiatry	22	4.7
4	Autism Research	Behavioral Sciences Psychology	19	4.7
5	Frontiers in neuroscience	Neurosciences & Neurology	19	5.3
6	Sensors	Engineering	19	3.9
7	PLOS ONE	Science & Technology	18	3.7
8	Biomedical Signal Processing and Control	Engineering	15	5.1
9	Brain Sciences	Neurosciences & Neurology	13	3.3
10	Journal of Autism and Developmental Disorder	Psychology	13	3.9

AI, artificial intelligence; ASD, autism spectrum disorder.

**Table 4:** Top 10 cited journals with publications on AI in ASD.

Rank	Cited journals	Research fields	Counts	Journal impact factor 2022
1	Journal of Autism and Developmental Disorders	Psychology	555	3.9
2	PLOS ONE	Science & Technology	361	3.7
3	Autism Research	Behavioral Sciences; Psychology	283	4.7
4	Neuroimage	Neurosciences &Neurology Radiology, Nuclear Medicine & Medical Imaging	280	5.7
5	Journal of Child Psychology and Psychiatry	Psychology; Psychiatry	224	7.6
6	Neuroimage-Clinical	Neurosciences & Neurology	195	4.2
7	Autism	Psychology	190	5.2
8	Molecular Autism	Genetics & Heredity Neurosciences& Neurology	185	6.2
9	Scientific Reports	Science & Technology	181	4.6
10	Frontiers in Human Neuroscience	Neurosciences & Neurology Psychology	174	2.9

AI, artificial intelligence; ASD, autism spectrum disorder.

### 3.5 Keywords

**Figure 6:**
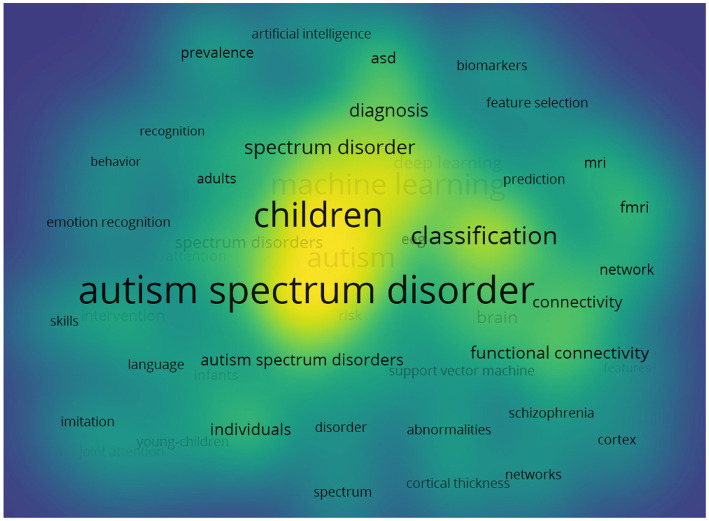
Cooperation of institutions that contributed to publications on AI in ASD. AI, artificial intelligence; ASD, autism spectrum disorder.

**Figure 7:**
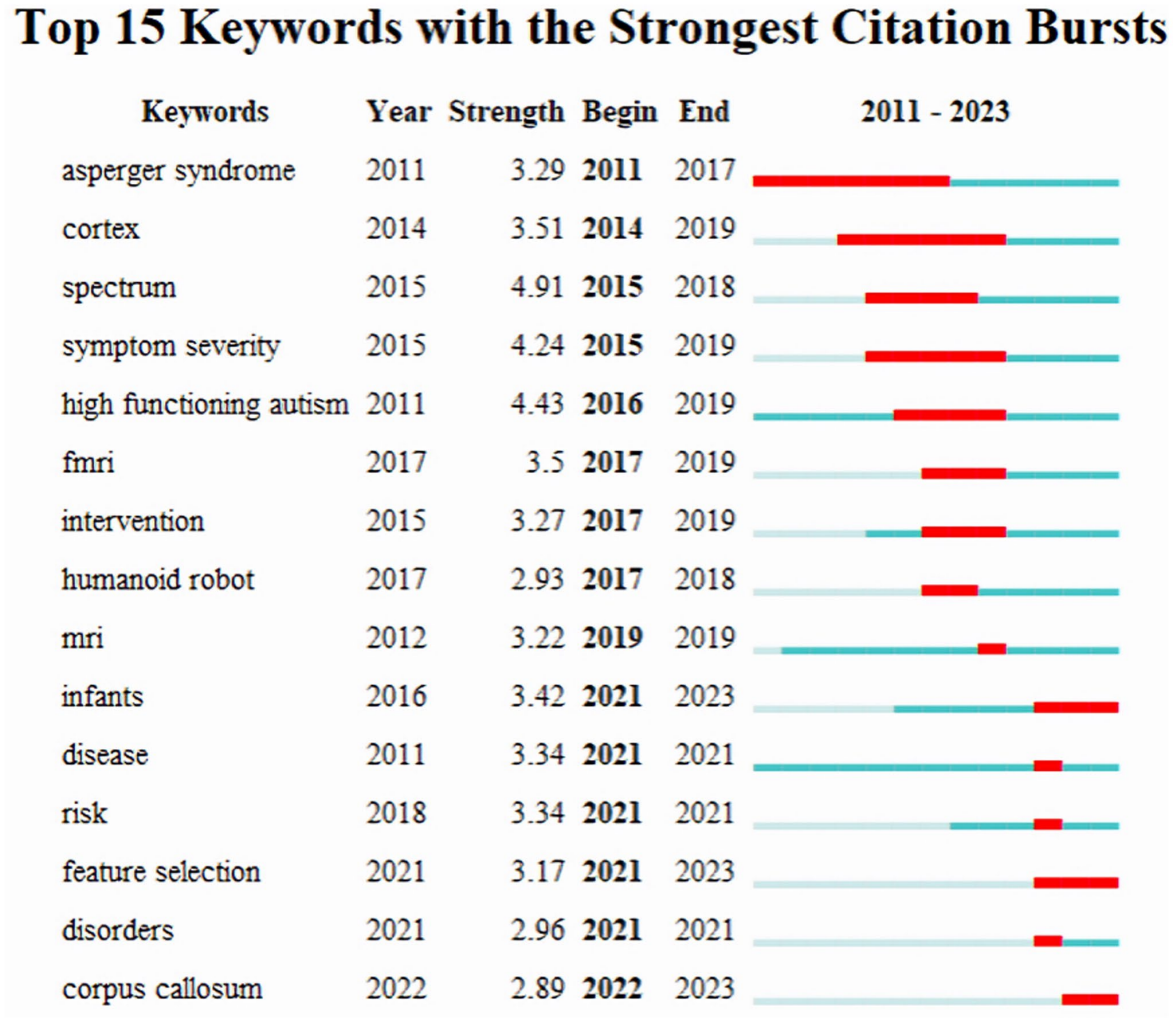
Dual-map overlay of journals that contributed to publications on AI in ASD. AI, artificial intelligence; ASD, autism spectrum disorder.

Based on keyword co-occurrence cooperation networks, a diagram of emerging keywords progressing over time was analyzed. The default settings of the VOSviewer software were used to analyze the hot keywords from the citations. As shown in Figure 6, a yellow block represents a keyword with higher frequency counts; those with lower frequency counts form the blue block. With a high number of occurrences, autism spectrum disorder (295), children (255), classification (156) and diagnosis (77) were the more active keywords used in the publications. The setting of CiteSpace was then switched to the following pattern: Year Per Slice = 1, Top N% = 8.0%, and Minimum Duration = 1, and the results of Figure 7. The keywords with the greatest outburst intensity were spectrum, high functioning autism, and symptom severity. The red squares represent emerging keywords for the timeline examined. Asperger syndrome and cortex were the keywords with the longest use, and infants, feature selection, and corpus callosum were the most prevalent keywords from 2021 to 2023.

### 3.6 Citing articles and references

**Figure 8:**
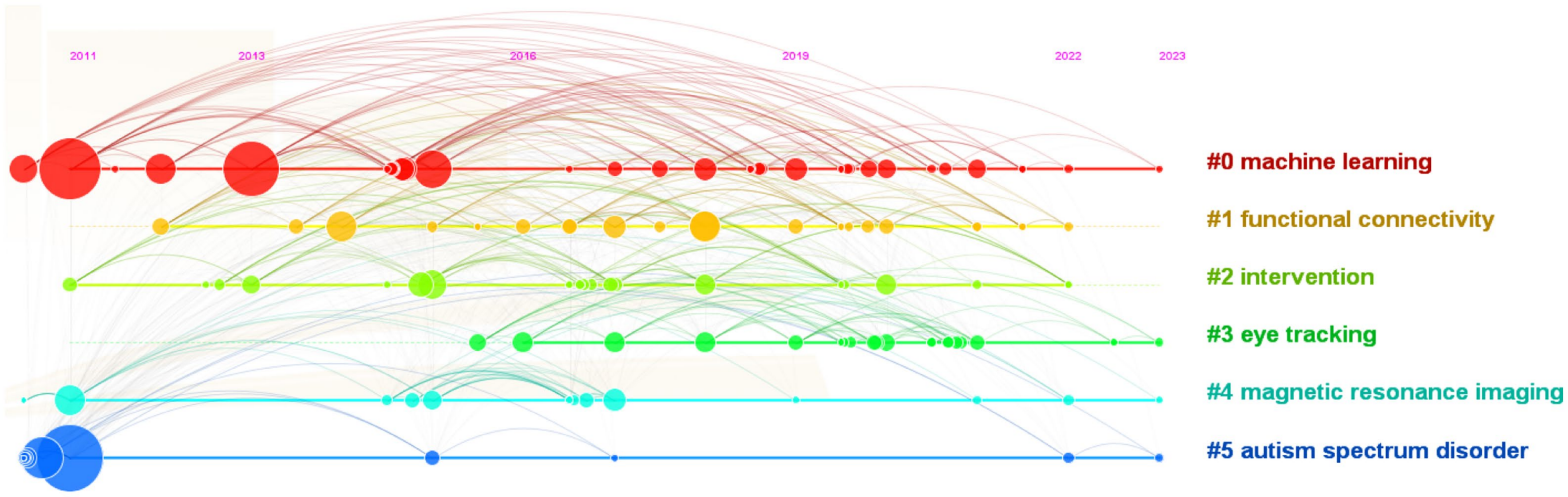
Density visualization of keywords in publications on AI in ASD. AI, artificial intelligence; ASD, autism spectrum disorder; fMRI, functional magnetic resonance imaging; MRI, magnetic resonance imaging.

The references had a close relationship to the research subjects. The co-cited references were clustered using the CiteSpace’s default settings, and label clusters with indexing terms were selected. The research frontiers of the cited references are represented by the cluster labels. In Figure 8, the sizes of the clusters found from the references are listed in ascending order. The largest cluster (label #0) machine learning was discovered over the longest duration of AI research in ASD, which was 2011–2023, and has been a long-running hotspot of research. Based on the number of citations in all databases. Table 5 shows the top 10 citing articles on AI in ASD. These 10 articles were all about the screening and diagnosis of ASD using AI, which were based on various learning algorithms and classifiers to study the genes, neuroimaging, EEG, and scoring scales of ASD. In summary, the application of AI technologies in ASD is promising.

**Table 5:** Top 10 citing articles on AI in ASD.

Rank	Title of citing articles	Doi	Times cited	Interpretation of the findings
1	Early brain development in infants at high risk for autism spectrum disorder (Hazlett et al., 2017)	10.1038/nature21369	580	This study used a deep learning algorithm based on brain surface information obtained by MRI in individuals aged between 6 and 12 months to predict the diagnosis of autism in high-risk children at 24 months, which showed high accuracy. The study demonstrates that early brain changes occur during the period when autistic behaviors first appear.
2	Identification of autism spectrum disorder using deep learning and the ABIDE dataset (Heinsfeld et al., 2018)	10.1016/j.nicl.2017.08.017	387	This study extracted patterns of functional connectivity that objectively identify ASD from functional brain imaging data based on the ABIDE dataset by deep learning models. The authors also identified the areas of the brain that contributed most to differentiating ASD from the anticorrelation of brain function between anterior and posterior areas of the brain.
3	EEG complexity as a biomarker for autism spectrum disorder risk (Bosl et al., 2011)	10.1186/1741-7015-9-18	292	This study used several machine learning algorithms to analyze the modified multiscale entropy computed on the basis of resting state EEG data, which may be a useful biomarker for early detection of risk for ASD in infants.
4	Genome-wide prediction and functional characterization of the genetic basis of autism spectrum disorder (Krishnan et al., 2016)	10.1038/nn.4353	222	The authors developed a machine-learning approach based on a human brain-specific gene network to show a genome-wide prediction of ASD risk genes, and they demonstrated that a large number of ASD genes converge in a few key pathways and developmental stages of the brain, as well as identified possible causative genes.
5	Functional neuroimaging of high-risk 6-month-old infants predicts a diagnosis of autism at 24 months of age (Emerson et al., 2017)	10.1126/scitranslmed.aag2882	195	This study applied a fully cross-validated machine learning algorithm based on functional brain connections of high-risk 6-month-old infants and showed a high predictive value of infants who received a diagnosis of ASD at 24 months.
6	Functional connectivity classification of autism identifies highly predictive brain features but falls short of biomarker standards (Plitt et al., 2015)	10.1016/j.nicl.2014.12.013	185	This study compared the classification accuracy of a machine learning classifier based on rs-fMRI data to a classifier based on scores on behavioral metrics for ASD, and the results suggested that individuals can be classified as having ASD from rs-fMRI scans alone but that this approach does not meet biomarker standards.
7	A small number of abnormal brain connections predicts adult autism spectrum disorder (Yahata et al., 2016)	10.1038/ncomms11254	167	This study developed a novel machine-learning algorithm that can identify a small number of functional connections separating adult ASD and typically developed individuals, and the classifier achieved high accuracy.
8	Use of machine learning to shorten observation-based screening and diagnosis of autism (Wall et al., 2012)	10.1038/tp.2012.10	151	This study used a series of machine learning algorithms based on the Autism Diagnostic Observation Schedule, and the results showed this method can shorten observation-based screening and diagnosis of autism.
9	Whole-genome deep-learning analysis identifies contribution of noncoding mutations to autism risk (Zhou et al., 2019)	10.1038/s41588-019-0420-0	128	This study applied a deep-learning-based framework in 1,790 ASD simplex families and revealed a convergent genetic landscape of coding and noncoding mutations in ASD.
10	Fuzzy Synchronization Likelihood-wavelet methodology for diagnosis of autism spectrum disorder (Ahmadlou et al., 2012)	10.1016/j.jneumeth.2012.08.020	125	This study presented a methodology using Fuzzy Synchronization Likelihood based on EEG and Neural Network classifier to diagnose ASD, which showed high accuracy.

ABIDE, autism brain imaging data exchange; AI, artificial intelligence; ASD, autism spectrum disorder; EEG, electroencephalography; MRI, magnetic resonance imaging; rs-fMRI, resting state functional magnetic resonance imaging.

## 4 Discussion

### 4.1 General data

Based on the results mentioned above, it is evident that the number of publications on AI in ASD has increased dramatically over the past 5 years, especially after 2020. This indicates that this field of research has gradually become popular in recent years. Machine learning and deep learning in computational intelligence-based systems, together with all of artificial intelligence’s auxiliary tools, are increasingly utilized in the medical field. Shahamiri et al. (2022) proposed a new classification system employing deep learning technology that can be integrated into current ASD screening to facilitate early detection of ASD features for stakeholders. As such, there have been numerous studies on AI in screening, diagnosis, and treatment of ASD (Lu and Perkowski, 2021; van den Berk-smeekens et al., 2022). Mertz (2021) summarized that AI, virtual reality, machine learning (ML), as well as other techniques have been used to advance ASD diagnosis and therapy. On the influence of national publications, the United States had the highest H-index, and England had the highest centrality. This indicates that studies from the two countries have heading positions in the research field. Additionally, China ranked second in the number of publications and H-index but had a lower centrality. Although India had fewer publications, its centrality and influence were strong. Viewed according to H-Index, Stanford University, the University of California System, and Harvard University had stronger impacts. This was noted in the analysis of national publication issuances. According to an evaluation of the journal’s research area, it is clear that research has concentrated on the application of AI technologies along with an understanding of ASD to create more useful systems to assist in the screening, diagnosis, and intervention of ASD. Particularly, the identification of ASD using AI is a popular research area. Specifically, based on MRI analysis, AI is widely used to identify ASD (Dekhil et al., 2018; Ahammed et al., 2021; Duan et al., 2022). In addition, these papers also reported AI has been used to identify ASD using specific genes (Lin et al., 2020; Tang et al., 2023) or perturbed metabolism (Graham et al., 2020; Zhang et al., 2023) or thermographic and EEG data (Haputhanthri et al., 2020). The clustering timeline of emerging keywords and co-cited references contains areas of current research focus and frontiers.

### 4.2 Research hotspots

Research hotspots were identified by analyzing emerging keywords. Different research hotspots are represented by the emerging keywords in the various periods.

The emerging keywords with the greatest outburst intensity were spectrum (4.91), high functioning autism (4.43), and symptom severity (4.24). Asperger’s syndrome was the keyword with the longest use. These keywords were all related to ASD: Asperger syndrome and high-functioning autism are subtypes of ASD, and spectrum is correlated to autism spectrum disorder, and symptom severity was emphasized. Unlike low-functioning autism, patients with Asperger syndrome and high-functioning autism both have normal intelligence, and these situations are more challenging to detect and diagnose, which may be why they are hotspots for research on AI in ASD. Bi et al. (2022) created a clustering-evolutionary random support vector machine ensemble (CERSVME) based on fMRI to identify Asperger syndrome that showed a high accuracy of 95.24%. Kimura et al. (2019) detected a candidate marker (cg20793532) annotated to the *PPP2R2C* gene, which offers a potential blood biomarker for the identification of high-functioning ASD, by employing two different ML algorithms for marker selection. Comparing the burst time of the keywords Asperger syndrome and high-functioning autism, the latter is gradually becoming a newer research hotspot. The keyword spectrum burst in 2015 may be attributed to the widespread use of the DSM-V, in which spectrum is emphasized (Volkmar and Reichow, 2013). The emerging keyword from 2015 to 2019 was symptom severity, which suggests researchers are not only focusing on the diagnosis of ASD but also the severity. Moradi et al. (2017) applied an ML method to predict the severity of ASD symptoms using measures of cortical thickness from aggregated data. Furthermore, they demonstrated the usefulness of the ABIDE database.

The emerging keyword from 2014 to 2019 was cortex and from 2017 to 2019 was fMRI. Cortex and fMRI are all relevant to neuropathology, which suggests that during this period researchers have begun to apply AI to the neuropathological study in ASD, especially neuroimaging study. Although some studies have shown changes in brain structure and function in ASD, credible brain biomarkers have not yet been found in ASD. The application of AI in this area enables the identification of ASD. Spera et al. evaluated the alteration of functional connections in male ASD children using multiple-site resting-state fMRI data optimized with ML. The results showed that functional connectivity (FC) is higher in ASD children between the angular gyrus and the right (R) hemisphere’s precuneus as well as between the right frontal operculum cortex and the left (L) hemisphere’s pars opercularis. Weaker connections in ASD are found between the L supramarginal gyrus and the L planum polare, as well as between the intra-hemispheric R temporal fusiform cortex and the R hippocampus (Spera et al., 2019). Yamagata et al. (2019) applied an ML method to determine resting-state functional connectivity modes based on MRI data and discovered that there was a distinct functional relationship between the right anterior cingulate cortex and right middle temporal gyrus in ASD, indicating this FC may provide a neurobiological marker for diagnosis.

The emerging keywords from 2021 to 2023 were infants and feature selection, and from 2022 to 2023 were corpus callosum. This suggests that the study foci are beginning to shift to earlier diagnosis in infants, feature selection is becoming popular for application in identifying ASD, and the corpus callosum is becoming a new target for the screening of ASD. Besides, “autism spectrum disorder (295),” “children (255),” and “classification (156)” denoted the top three keywords in the included literature, which corroborate with the emerging keywords-infants and feature selection, all of which indicate that younger children are the hot population for research in this field, and that the application of AI in the screening and classification of ASD is popular. Alvari et al. (2021) used an ML model to identify micro-expressions in autistic and typical infants in their initial ecological interactions between the ages of 6 and 12 months. Vabalas et al. (2019) reviewed studies that have applied ML to predict autism in non-autistic individuals and showed that in contrast to parameter tuning, feature selection significantly contributes to bias. Sharif and Khan (2022) proposed a framework based on ML for the automated detection of ASD by extracting features from the corpus callosum and intracranial brain volume; their results showed that their framework attained excellent recognition accuracy and decreased the complication of the ML model through feature selection. With the rise in popularity of AI and the continued implementation of technologies for ASD, objective detection indicators of ASD are expected to be discovered. Furthermore, intervention and treatment of ASD can benefit from AI.

### 4.3 Limitations

This study has some limitations. First, there were restrictions on publication selection. Publications were downloaded only from the WoSCC database; the language was restricted to English, and the type of publication was limited to article. These criteria may have resulted in publication bias. Second, despite efforts made to minimize bias, no study method could fully reduce the biases. Third, the top 10 citing publications’ limitations were summarized, it was found that they fall into the following sections: (1) more than half of these publications investigated the use of AI technology to analyze MRI data to identify ASD, but these methods fall short of biomarker standards; (2) most models employed a dataset with a small examination population or a small amount of imaging data; and (3) AI identification and diagnosis is still in the preliminary stages, and few studies using AI technology to intervene in ASD have been conducted.

More types and larger datasets are required to develop reliable and usable screening and diagnostic systems. For example, collecting data on ASD screening across races, countries, and regions (Haque et al., 2021; Lu and Perkowski, 2021; Pietrucci et al., 2022) should be pursued. Additionally, more clinicians with different training levels should be engaged in screening the dataset and researching the algorithm to achieve a clinically valid diagnosis. Finally, the utilization rate of AI models can be further improved by enlarging the clinical samples in the validation system and using statistical methods to get great sensitivity and specificity.

## 5 Conclusion

This study conducted a systematic bibliometric and visualized analysis of studies in AI used in ASD, providing a comprehensive demonstration of the current state of research in this field. The analysis of number of publications and cooperation in countries, institutions, and journals, enables researchers to have an overall understanding of research in this area and to obtain countries, institutions, and journals that need to be prioritized and need to be strengthened for cooperation. Especially, the analysis of keywords and references of publications in this field presents the latest research hotspots and trends, providing directions for researchers.

In summary, the application of AI technologies to recognize, diagnose, and intervene in patients with autism spectrum disorders has become one of the most relevant applications of AI technologies, especially in recent years. “Infants,” feature selection, and corpus callosum have come to the forefront of research in this area. In the future, the use of ML methods combined with feature selection to identify ASD will potentially provide an objective diagnostic indicator. Further studies of the corpus callosum may provide biological indicators for ASD identification. The top 10 citing articles are all about the identification and diagnosis of ASD. They used deep learning, machine learning and other algorithms to make early prediction based on different data, including MRI information, brain functional connectivity, gene networks, EEG, behavioral scales, etc. Each model achieved high accuracy, suggesting that the analysis of relevant data based on AI algorithms such as machine learning can identify ASD early, possibly provide biomarkers, and further change the diagnostic patterns. Research on the use of AI in ASD is ongoing globally, with England being the most authoritative country in this area.

The application of AI in ASD has revolutionized clinical practice, particularly in the diagnosis and classification of ASD. More precise diagnosis, classification, and assisted remote services are made possible by this technology. In particular, based on MRI, brain connectivity, EEG and other data, using various machine learning algorithms to create diagnostic models for ASD, is a more extensive research content in this field. It is also a more mature part of AI used in ASD, which may change the current behavioral diagnosis of ASD, and may help identify ASD early and improve prognosis. However, these approaches have certain limitations. For instance, although it has been demonstrated that some training models have great sensitivity and specificity, their validity is debatable, and institutions frequently do not accept them for use in actual clinical work. In addition, although intervention has emerged in burst keywords in recent years, studies on AI used in the intervention of ASD are still limited. To overcome the current restrictions, more racial and national data are needed to educate and evaluate the system. Clinicians from various regions and with various levels of experience must be included, besides the computer scientists who are chiefly responsible for building the systems.

## Data availability statement

The original contributions presented in the study are included in the article/supplementary material, further inquiries can be directed to the corresponding authors.

## Author contributions

QJ: Formal analysis, Methodology, Writing – original draft. XW: Formal analysis, Writing – original draft, Methodology, Conceptualization. RZ: Software, Writing – original draft. BM: Writing – review & editing, Methodology. FF: Formal analysis, Writing – original draft. HH: Writing – review & editing, Methodology.
